# Cost-effectiveness of tiotropium versus usual care and glycopyrronium in the treatment of chronic obstructive pulmonary disease in Sweden

**DOI:** 10.1186/s12962-015-0040-1

**Published:** 2015-08-19

**Authors:** Oskar Eklund, Faraz Afzal, Fredrik Borgström

**Affiliations:** Quantify Research AB, Stockholm, Sweden; Boehringer Ingelheim Norway KS, Asker, Norway

**Keywords:** COPD, Exacerbations, Tiotropium, Glycopyrronium, Cost-effectiveness, Markov cohort model

## Abstract

**Background:**

Tiotropium (TIO) is a well-established bronchodilator, LAMA (long-acting anticholinergic), for the treatment of moderate to very severe chronic obstructive pulmonary disease (COPD). Clinical evidence suggests that tiotropium is superior to usual non-LAMA care (UC) but may also have benefits compared to other LAMAs in preventing and limiting the effects of severe exacerbations. The primary objective of this study was to undertake a cost-effectiveness analysis of adding tiotropium to usual care versus usual care alone. A secondary objective was to assess the cost-effectiveness of tiotropium compared to glycopyrronium (GLY), another LAMA. The study was conducted with a Swedish setting in mind.

**Methods:**

A Markov cohort model, incorporating the effects of exacerbations, was populated with efficacy data from the UPLIFT and SPARK trials and epidemiological data relevant for a Swedish patient population. Treatment efficacy of tiotropium was modelled as a lowering of the risk of exacerbations and as a slow-down of overall disease progression. The model followed patients over their remaining life-time.

**Results:**

The base case analysis showed that patients treated with tiotropium gained 0.07 quality-adjusted life years (QALYs) compared to usual care alone at an incremental cost of SEK 15,041, resulting in a cost per QALY gained of SEK 224,850. Compared to glycopyrronium the QALY gained was estimated to 0.23 QALYs in favour of tiotropium at an incremental cost of SEK 2423, yielding a cost per QALY gained of SEK 10,456. The results were mainly driven by differences in the risk of severe exacerbations.

**Conclusion:**

At the current implicit willingness-to-pay (WTP) per QALY threshold in Sweden, the results from this study indicate that tiotropium is a highly cost-effective intervention when added to usual non-LAMA care in the treatment of moderate to very severe COPD in Sweden. In addition, tiotropium is a highly cost-effective intervention when compared to glycopyrronium monotherapy.

## Background

Chronic obstructive pulmonary disease (COPD) is one of the most common chronic diseases worldwide and it is a major cause of morbidity and mortality. The prevalence of COPD in the total adult population is reported to be in the range of 5–10 % [1–4]. This rather high prevalence translates into a high burden of illness. A study measuring costs of COPD in Sweden in 2010, estimated the total costs of COPD to be €1.5 billion annually [5]. COPD exacerbations, which are defined as “acute and sustained worsening of the patient’s respiratory symptoms beyond normal day-to-day variations that result in an increased need for medication […]”, are important drivers of COPD-related costs [6]. Studies from the US and the UK have shown that exacerbations are the most common reasons for hospitalisation of COPD patients [7, 8] and that hospitalisations, in turn, stand for 54 % of direct COPD costs [9]. Furthermore, exacerbations have been shown to increase mortality [10, 11] and decrease health-related quality of life [12, 13] in COPD patients.

Pharmacologic therapy for COPD include inhaled corticosteroids (ICS) and long-acting bronchodilators such as LABAs (long-acting β_2_-agonists) or LAMAs (long-acting anticholinergics), which are recommended therapies for patients with moderate to very severe COPD [6]. In Sweden monotherapy with LAMAs (e.g. tiotropium or glycopyrronium) is the first-line treatment. Alternatively, LABAs (e.g. salmeterol or indacaterol) can be considered, either as monotherapy or as add-on therapy [14]. LAMA/LABA combination inhalers (e.g. glycopyrronium + indacaterol) are available, but have restricted reimbursement to patients who have not achieved adequate effect from monotherapy [15]. From a Swedish perspective, combination therapies are therefore not relevant comparators to LAMA and LABA monotherapies. Inhaled corticosteroid therapy is only considered in combination with LABA therapy for patients with severe or very severe COPD [14].

Numerous studies have shown that regular use of inhaled LAMAs, LABAs and corticosteroids can lower the incidence and effect of exacerbations [16]. In a 4-year, randomized, double-blind trial (UPLIFT) with 5993 patients, tiotropium significantly improved lung function, in terms of forced expiratory volume in 1 s (FEV_1_), and lowered the risk of exacerbations when added to usual non-LAMA care (placebo), compared to usual non-LAMA care alone [17].

Evidence from recent studies also suggest that tiotropium is superior to both once-daily [18] and twice-daily [19] administered LABAs in preventing exacerbations. Furthermore, in a recent head-to-head trial (SPARK), patients treated with tiotropium had lower rates of severe exacerbations (i.e. those leading to hospitalisation) than patients treated with glycopyrronium [20]. All in all, the evidence to date suggests that tiotropium is superior to usual non-LAMA care (UPLIFT) in terms of preserving overall lung function and in preventing exacerbations, but also that tiotropium performs better than glycopyrronium (SPARK) in terms of preventing severe exacerbations. Since exacerbations are key drivers of costs and morbidity in COPD, this new data ensures a more valid comparison of existing treatment alternatives.

Even though a treatment shows a positive clinical profile it is important to ensure that the potential gains in terms of improved health for the patient come at an acceptable cost to the payers of health care. Today, the standard approach to analyse this is by performing a cost-effectiveness analysis. Tiotropium has been shown to be a cost-effective treatment compared to usual care in the management of COPD in a number of countries [21]. However, such an assessment has not been conducted for Sweden. As such, the main objective of this study was to carry out a cost-effectiveness analysis on adding tiotropium to usual care versus usual care alone from a Swedish perspective. Furthermore, evidence on the cost-effectiveness of tiotropium versus other LAMAs is scarce. However, with the publication of the SPARK trial, comparing tiotropium and glycopyrronium head-to-head, such an analysis has now become possible. As such, a secondary objective was to assess the cost-effectiveness of tiotropium compared to glycopyrronium based on the SPARK trial.

### UPLIFT and SPARK trials

UPLIFT (ClinicalTrials.gov, NCT00144339) [17] was a randomised, double-blind, placebo-controlled study, investigating the efficacy of tiotropium when added to non-LAMA usual care (placebo) compared to placebo alone. A total of 5993 patients aged 40 years or more (mean age 65 ± 8 years), with a forced expiratory volume in 1 s (FEV_1_) of 70 % or less after bronchodilation and a ratio of FEV_1_ to forced vital capacity (FVC) of 70 % or less, were studied over a 4 year period. Co-primary end points were the rate of decline in mean FEV_1_ before and after bronchodilation beginning on day 30. Secondary end points were, among other things, rates of exacerbations, measures of FVC, changes in St. George’s Respiratory Questionnaire (SGRQ) responses (a disease-specific health-related quality of life instrument) and mortality. The main findings were that mean absolute improvements in FEV_1_ in the tiotropium group were maintained throughout the trial as compared to placebo (P < 0.001). However, after day 30, the differences between tiotropium and placebo in terms of the rate of mean FEV_1_ decline before and after bronchodilation were not significant. Another important result, was that the number of exacerbations per patient-year declined from 0.85 to 0.73 (P < 0.001) in favour of tiotropium. However, the difference was not significant for severe exacerbations (0.15 in the tiotropium group versus 0.16 in the placebo group) (P = 0.34) [17].

SPARK (ClinicalTrials.gov, NCT01120691) [20] was a randomised parallel group study investigating the relative efficacy of QVA149 (glycopyrronium and indacaterol combination) versus glycopyrronium monotherapy and tiotropium monotherapy. 2224 patients were enrolled, aged 40 years or more, with severe or very severe COPD, and one or more moderate COPD exacerbations in the last year. Results showed that there was no significant difference between glycopyrronium and tiotropium in preventing mild (GLY/TIO RR: 0.99, P = 0.90) and moderate or severe (GLY/TIO RR: 1.03, P = 0.68) exacerbations. However, in the case of severe exacerbations alone, tiotropium was superior to glycopyrronium (GLY/TIO RR: 1.43, P = 0.025). This is the relative treatment effect of tiotropium versus glycopyrronium used in the model.

## Methods

### COPD models

A majority of the cost-effectiveness models in the COPD literature are Markov cohort models [6, 22]. These types of models are well-suited for modeling a chronic and slowly progressing disease like COPD. Typically, model health states are constructed around the established Global Initiative for Chronic Obstructive Lung Disease (GOLD) classification of severity of airflow limitation [23]. This classification is based on four groups; mild (GOLD I FEV_1_ ≥ 80 %), moderate (GOLD II 50 % ≤ FEV_1_ < 80 %), severe (GOLD III 30 % ≤ FEV_1_ < 50 %) and very severe (GOLD IV FEV_1_ < 30 %) COPD, each representing an interval of post-bronchodilator FEV1, a measure of airflow [23]. COPD develops late in life and its onset is slow. As a result, several published models, e.g. [24, 25], assume that the cohort begins in the more severe GOLD states, where pharmacological therapies are introduced. Some models, e.g. [26, 27], also incorporate costs and effects of exacerbations. How these events are incorporated differs from model to model, but a common practice is to model exacerbations as events occurring within the health (GOLD) states. This makes it possible to add baseline costs and outcomes associated with each health state as well as additional event-specific costs and outcomes associated with exacerbations.

### Model description

**Fig. 1:**
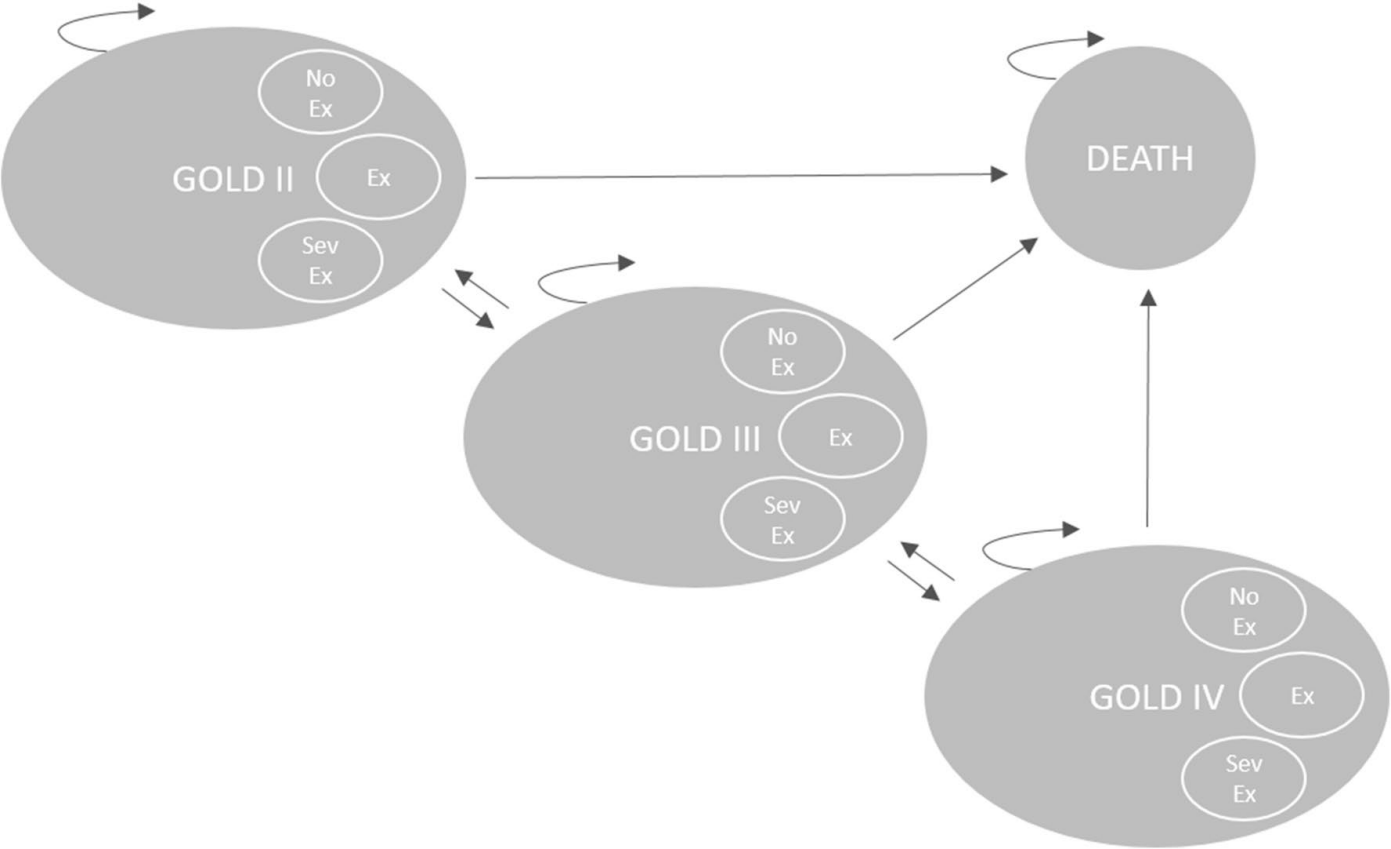
Markov model state transition diagram. Within each state a patient can experience any one of the following events: (1) no exacerbation (NoEx), (2) non-severe exacerbation (Ex), (3) severe exacerbation (SevEx)

The Markov cohort model, shown in Fig. 1, was built in Treeage Pro Suite 2014. It was based on four health states (GOLD II, GOLD III, GOLD IV and DEATH), between which the cohort of patients could move with certain probabilities. Patients were modelled from the age of treatment initiation (65 years old) until they deceased or reached the age of 100. Three cohorts were analysed, one receiving usual non-LAMA care similar to the placebo arm in the UPLIFT trial and two receiving additional treatment with either tiotropium 18 µg (Spiriva HandiHaler) or glycopyrronium 44 µg (Seebri Breezhaler). Upon entering the model, all patients were distributed across the separate states according to the observed distributions found in the UPLIFT trial (GOLD II: 48 %, GOLD III: 44 %, GOLD IV: 8 %) [17]. Within each three month cycle the patients could then experience any one of the following three events; (1) no exacerbation (2) non-severe exacerbation or (3) severe exacerbation. Each event occurred with certain state-specific probabilities (risks). Based on trial data from UPLIFT and SPARK, these probabilities were varied across the treatment alternatives in order to reflect relative efficacy of the treatment alternatives. Each state and event combination had different costs and effects associated with that particular combination. Subsequent to each event, the patients could either die or transition to one of the four health states for a new three month cycle to begin. The transition probabilities were varied across treatment alternatives based on trial data from UPLIFT. In this way, a more effective treatment acted to slow down the overall progression of disease.

### Target patient population

The base case model population was assumed to be similar to that of the UPLIFT trial in terms of age (65 years), disease state (GOLD II–IV), risks of exacerbations and the usual care received (non-LAMA). In addition, to accurately reflect the real world characteristics of Swedish COPD patients, the model was populated with mortality data representative for a Swedish patient population.

### Clinical data

Data concerning treatment efficacy, i.e. transition probabilities and relative risks of exacerbations, were mainly obtained from the Hettle et al. [28], who in turn derived estimates from UPLIFT [17]. Glycopyrronium was assumed to be equivalent to tiotropium in terms of effect on overall lung function (FEV_1_). The rationale for this assumption is well-founded; recent trials (GLOW1-2) [29] and indeed SPARK [20], as well as network meta analyses [30, 31], have shown comparability in terms of the effect on FEV_1_ between tiotropium and glycopyrronium and other LAMAs. Thus, efficacy data (FEV_1_) from the tiotropium arm of the UPLIFT trial were used to model disease progression in both the tiotropium and the glycopyrronium cohorts. However, data on relative risks of exacerbations for tiotropium versus glycopyrronium, were obtained from the SPARK trial. Table 1 provides an overview of the sources used to model treatment efficacy.

**Table 1:** Source overview of clinical inputs

Clinical input	Usual care	Tiotropium	Glycopyrronium
Transition probabilities			
Cycles on treatment (year 0–4)	UPLIFT (UC arm)	UPLIFT (TIO arm)	UPLIFT (TIO arm)^a^
Cycles off treatment (year 4–>)	UPLIFT (UC arm)	UPLIFT (UC arm)^b^	UPLIFT (UC arm)^b^
Probabilities of exacerbations			
Baseline risks of exacerbations	UPLIFT (UC arm)	UPLIFT (TIO arm)	UPLIFT (TIO arm)
Relative risks	1.00	N/A (probs applied)	SPARK RR TIO/GLY^c^

^a^Several trials have shown comparable efficacy between tiotropium and glycopyrronium in terms of overall lung function (FEV_1_)

^b^As there is little persistence in the effect of LAMAs after stopping treatment, transition probabilities were assumed to return to the placebo arm (usual care) probabilities in the cycle after stopping treatment

^c^Applied to baseline risks of exacerbations from UPLIFT (TIO arm)

Hettle et al. [28] derived sets of transition probabilities for tiotropium and usual care arms using data from UPLIFT. A separate set of transition probabilities were obtained for the first cycle on treatment, in order to capture the initial 30-day effect of tiotropium on FEV_1_ [28]. In the base case analysis, patients were assumed to be on treatment for 4 years from the start of simulation, based on the observed treatment duration in UPLIFT [17]. The assumption was made to ensure that results were not driven by unrealistic assumptions about treatment duration (some share of patients will in practice discontinue treatment). As there is little persistence in the effect of LAMAs after stopping treatment, transition probabilities were assumed to return to the placebo arm (usual care) probabilities from UPLIFT in the cycle after stopping treatment. The sets of transition probabilities used in the model are shown in Table 2 below.

**Table 2:** Model transition probabilities by GOLD states

Usual care	First cycle			Subsequent treatment cycles		
	TO			TO		
	GOLD II	GOLD III	GOLD IV	GOLD II	GOLD III	GOLD IV
FROM						
GOLD II	0.86	0.13	0.01	0.91	0.09	0.00
GOLD III	0.13	0.81	0.06	0.08	0.88	0.04
GOLD IV	0.02	0.22	0.76	0.00	0.13	0.87

Tiotropium/glycopyrronium	First cycle			Subsequent treatment cycles		
	TO			TO		
	GOLD II	GOLD III	GOLD IV	GOLD II	GOLD III	GOLD IV
FROM						
GOLD II	0.92	0.08	0.00	0.92	0.08	0.00
GOLD III	0.17	0.80	0.03	0.08	0.88	0.04
GOLD IV	0.03	0.28	0.69	0.00	0.12	0.88

Source: [28] (derived from [17]). For tiotropium and glycopyrronium cohorts, usual care probabilities were assumed to hold when off treatment. Probabilities have been recalculated to reflect three month probabilities. Death has been excluded, as this model carries separate mortality rates derived for a Swedish population

Rates of exacerbations for usual care and tiotropium cohorts were obtained from Hettle et al. [28] (derived from UPLIFT [17]) and recalculated to reflect 3 month risks. Relative risks of exacerbations for tiotropium versus glycopyrronium were obtained from SPARK.

Table 3 shows the risks of different types of exacerbations by GOLD state for the different treatment alternatives. In sum, the efficacy of tiotropium versus usual care was modelled as a lowering of the risk of exacerbations (Table 3) and as a delay in the overall progression of COPD (Table 2). The efficacy of tiotropium against glycopyrronium was modelled solely based on the lower risk of severe exacerbations (Table 3) found in SPARK.

**Table 3:** Model probabilities of exacerbations by GOLD states

GOLD	Usual care			Tiotropium			Glycopyrronium		
	II	III	IV	II	III	IV	II	III	IV
No exacerbation	0.84	0.78	0.73	0.87	0.81	0.77	0.86	0.79	0.74
Non-severe exacerbation	0.14	0.17	0.18	0.11	0.14	0.15	0.11	0.14	0.14
Severe exacerbation	0.02	0.05	0.08	0.02	0.05	0.08	0.03	0.07	0.12

Source: [28] (derived from [17]), [20] and own calculations to reflect three month probabilities

### Epidemiological data

Age-differentiated mortality rates were derived for each GOLD state (II–IV). Excess mortality rates associated with events were derived in the case of severe exacerbations, but not in the case of “non-severe exacerbations” or “no exacerbations”, for which the state-specific mortality rates were assumed to hold.

Mortality rates for health states and severe exacerbations were comprised of two separate parts; (1) baseline age-differentiated mortality for the general population in Sweden in 2012, and (2) excess mortality associated with the state or event in question. Baseline age-differentiated mortality for the general population in 2012 was obtained from the National Board of Health and Welfare (Socialstyrelsen) and its database on causes of deaths [32]. From the database it was possible to discern the share of total deaths that were related to COPD (ICD-10: J44) and to factor these out of the baseline mortality rates. The corrected and age-differentiated mortality rates for the general population were then used as baseline rates, on top of which excess mortality rates were added. Point estimates for excess mortality associated with each GOLD state were obtained from Garcia-Aymerich et al. [10] and extrapolated over age intervals using the change in relative risk between age groups from Hoogendoorn et al. [33]. Similarly, age-differentiated excess mortality rates for severe exacerbations were generated using point estimates and changes in relative risks between age groups from Hoogendoorn et al. [33].

### Health economic data

The analysis takes on a societal perspective. However, because the start age was set to 65 years in the base case, i.e. equal to the retirement age in Sweden, indirect costs were deemed to be negligible and thus only direct costs were considered in this model. Direct costs were assigned for the different state-event combinations using data from two different studies of Swedish COPD patients [5, 34]. Three month COPD maintenance costs, e.g. costs of drugs, outpatient care, oxygen therapy and other non-exacerbation related direct costs, were obtained from Jansson et al. [5]. Costs of non-severe and severe exacerbations, from Andersson et al. [34], were added to the maintenance costs to obtain the total cost for each state-event combination. Table 4 shows the direct costs per cycle used in the model.

**Table 4:** Direct costs (SEK 2014) by GOLD state and type of event

	GOLD II	GOLD III	GOLD IV
No exacerbation	1284	3032	4297
Non-severe exacerbation	4423	6170	7436
Severe exacerbation	27,817	29,564	30,830

Source: maintenance costs by GOLD-state from [5], exacerbation costs from [34]. Costs for mild and moderate exacerbations were merged to form non-severe exacerbations

As in the case of costs, separate utility weights were assigned to the different state-event combinations. The baseline utility weights for each GOLD state were obtained from a Swedish study, Ståhl et al. [35]. Decrements associated with non-severe exacerbations (1.66 %) and severe exacerbations (4.82 %) were obtained from Hoogendorn et al. [33]. The decrements were applied to the annual utility weights, thus making it a conservative assumption of the utility loss of exacerbations. Table 5 shows the annual utility weights associated with each state-event combination.

**Table 5:** Utility weights by GOLD state and type of exacerbation

	GOLD II	GOLD III	GOLD IV
No exacerbation	0.73	0.74	0.52
Non-severe exacerbation	0.72	0.73	0.51
Severe exacerbation	0.69	0.70	0.49

Source: baseline utility weights (no exacerbation) [35], annual utility decrements [33]

### Model validation

The model was thoroughly validated. Cohort transition matrices for the distribution of GOLD states as well as the predicted risks of exacerbations were reprogrammed in Excel and compared with similar outputs in the TreeAge model. Both approaches showed the same results. The annualised rates of severe exacerbations predicted by the model in a 4 year simulation (the length of UPLIFT) were very close to those of Hettle et al. [28] (Hettle et al. SevEx: UC, 0.22; TIO, 0,21—Cohort model SevEx: UC, 0.24; TIO, 0.22). The difference can be explained by different mortality rates used in our model compared to the Hettle et al. [28] model.

### Base case analysis

In base case, cost-effectiveness of tiotropium was compared to usual care and to glycopyrronium. The assumptions made in the base case analysis are shown in Table 6.

**Table 6:** Base case inputs and assumptions

Variable	Description	References
Time horizon	Life time	Assumption
Start age	65 years	Based on [17] (mean)
Sex	Both	Assumption
Treatment duration	4 years	Based on [17] length
Cost of tiotropium	SEK 12.77 per day	[36]
Cost of glycopyrronium	SEK 10.48 per day	[36]
Discount rate	3 % per annum (both effects and costs)	[37]
Initial prob	GOLD II: 48 %, GOLD III: 44 %, GOLD IV: 8 %	[17]
Transition prob	Probabilities (3-month)	[28]
Mortality states	GOLD, age and sex dependent	[10, 32, 33]
Mortality severe ex	Excess mort increasing with age	[33]
Exacerbation risks	Probabilities (3-month)	[20, 28]

### Sensitivity analysis

Uncertainty was assessed both through deterministic and probabilistic sensitivity analysis (PSA). Extensive one-way deterministic sensitivity analyses were performed on key model parameters, in order to assess their impact on the main result (ICER). Different subgroups of interest were also assessed, e.g. males and females only as well as patients with moderate, severe and very severe COPD respectively.

Probabilistic sensitivity analysis was performed relative to the two comparators separately using Monte Carlo simulation (10,000 iterations). Distributions were assigned to key model inputs; treatment effect on exacerbations (lognormal), direct costs (uniform) and utility weights (uniform). The uncertainty of the treatment effect on non-severe and severe exacerbations was assessed using the 95 % confidence intervals from the respective studies. Uncertainty of direct costs and utility weights were modelled using a uniform distribution and ±20 % interval around the point estimates.

## Results

### Base case

Base case results from the comparison between tiotropium and usual care are presented in Table 7. Tiotropium added 0.07 QALYs and 0.08 life years compared to usual care alone. In addition, tiotropium increased total costs by SEK 15,041 over the lifetime of the average patient. The resulting incremental cost-effectiveness ratio (ICER) was SEK 224,850.

**Table 7:** Base case results—tiotropium vs usual care

	UC	TIO	TIO–UC
Costs (SEK 2014)			
Treatment costs	0	17,315	17,315
Direct costs	167,654	165,380	−2274
Total costs	167,654	182,695	15,041
Health outcomes			
QALYs	7.18	7.25	0.07
Life years	10.18	10.26	0.08
ICER			224,850

All costs and effects discounted at an annual rate of 3 %

Base case results from the comparison between tiotropium and glycopyrronium are presented in Table 8. Tiotropium added 0.23 QALYs and increased total costs by SEK 2423 compared to glycopyrronium. The resulting ICER amounted to SEK 10,456.

**Table 8:** Base case results—tiotropium vs glycopyrronium

	GLY	TIO	TIO–GLY
Costs (SEK 2014)			
Treatment costs	13,965	17,315	3351
Direct costs	166,308	165,380	−928
Total costs	180,272	182,695	2423
Health outcomes			
QALYs	7.02	7.25	0.23
Life years	9.93	10.26	0.33
ICER			10,456

All costs and effects discounted at an annual rate of 3 %

### Sensitivity analysis

#### Deterministic sensitivity analysis

**Fig. 2:**
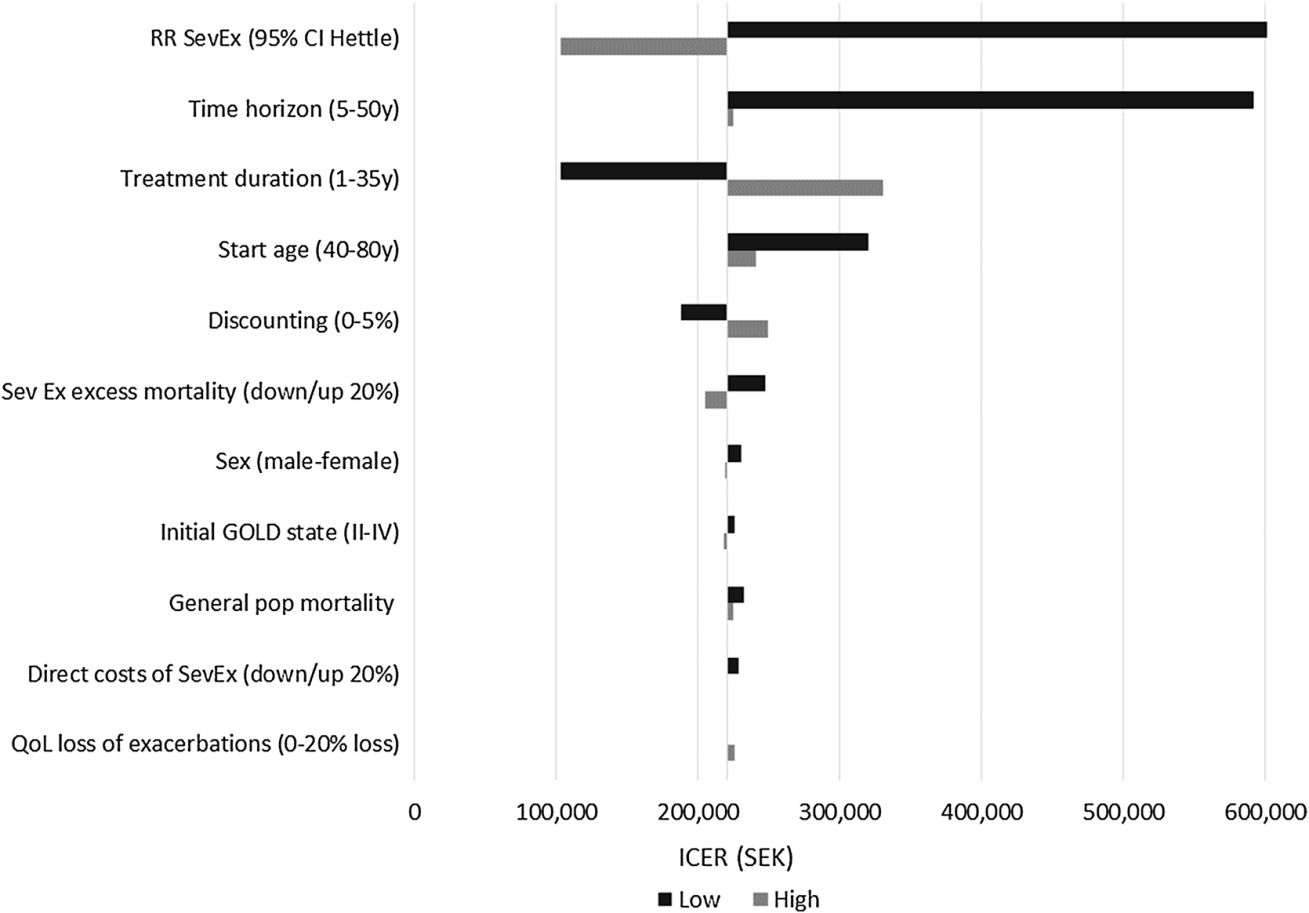
Tornado diagram ICER (TIO vs UC)

**Fig. 3:**
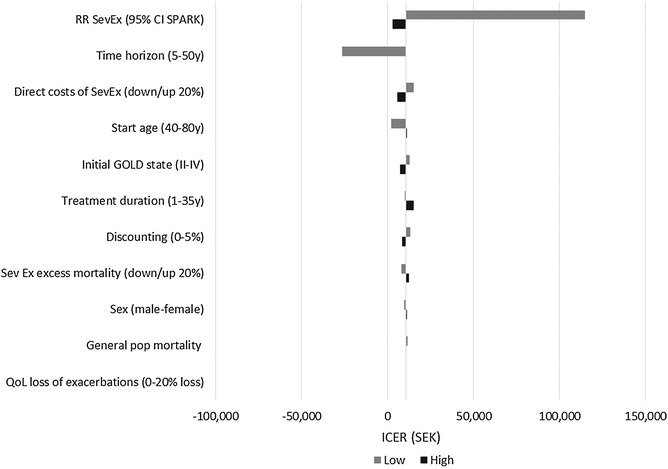
Tornado diagram ICER (TIO vs GLY)

Figures 2, 3 and Table 9 show how the ICER was affected when varying key model parameters one at a time. The parameters that impacted the ICER the most were time horizon and the treatment effect on severe exacerbations (RR SevEx). In a realistic scenario with a life-long time horizon, the treatment effect on severe exacerbations was by far the most important parameter. In the case of tiotropium versus usual care, applying the 95 % CI lower limit for the rate of severe exacerbations from Hettle et al. [28] substantially increased the ICER. In the case of tiotropium versus glycopyrronium, applying the 95 % CI lower limit for RR SevEx 1.05 (base case 1.43) increased the ICER to SEK 115,000, which would still be considered highly cost-effective. In fact, tiotropium remains cost-effective at current willingness-to-pay thresholds (SEK ~ 600,000) even when RR SevEx is as low as 1.02–1.03.

**Table 9:** One-way sensitivity analysis (ICER in SEK 2014)

Variable of interest	Value	TIO vs UC	TIO vs GLY
Base case		224,850	10,456
Discount rate	0 %	187,684	13,206
	5 %	249,658	8523
Time horizon	5 years	592,149	Dominating
	10 years	326,192	2233
	20 years	235,715	9641
Treatment duration	1 year	102,727	9849
	10 years	294,652	12,081
	Life (35 years)	330,299	14,962
Sex	Males only	230,862	9646
	Females only	220,021	11,141
Start age	40	320,470	2119
	80	240,894	11,043
GOLD start dist	All start in GOLD II	225,925	12,878
	All start in GOLD III	224,903	9172
	All start in GOLD IV	217,926	7084
Mortality	Normal mortality (not adjusted for COPD) for all GOLD states	232,402	11,522
	20 % higher excess mortality (Sev Ex)	204,979	12,424
	20 % lower excess mortality (Sev Ex)	247,036	8093
Effect of tiotropium on Sev Ex	Low 95 % CI from studies^a^ (RR Sev Ex)	103,232	114,589
	High 95 % CI from studies^a^ (RR Sev Ex)	5,845,054	2859
Direct costs of Sev Ex	20 % higher	221,336	5665
	20 % lower	228,367	15,246
QoL loss of Sev Ex exacerbations	0 % loss	225,934	10,528
	20 % loss	221,429	10,235

“*Dominating*” tiotropium is dominating, “*dominated*” tiotropium is dominated

Base case: RR SevEx GLY vs TIO = 1.43

^a^TIO vs UC, Hettle et al.; TIO vs GLY, RR SevEx in SPARK 1.43 (CI 1.05–1.97, P: 0.025)

### Probabilistic sensitivity analysis

**Fig. 4:**
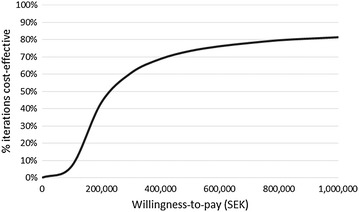
Cost-effectiveness acceptability curve for TIO vs UC

**Fig. 5:**
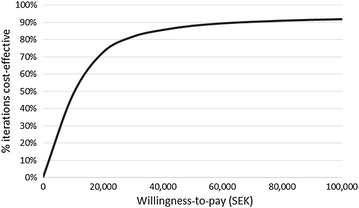
Cost-effectiveness acceptability curve for TIO vs GLY

Figures 4 and 5 show the cost-effectiveness acceptability curves for both comparators relative to tiotropium. Tiotropium is highly cost-effective (TIO vs UC: 80 %/TIO vs GLY: 90 %) at the implicit willingness-to-pay threshold for Sweden (SEK ~ 600,000).

## Discussion

The primary objective of this study was to assess the cost-effectiveness of tiotropium when added to usual non-LAMA care compared to usual care alone in a population relevant for a Swedish setting. The secondary objective was to compare the cost-effectiveness of tiotropium relative to glycopyrronium under similar circumstances. Tiotropium was found to be highly cost-effective relative to both comparators at current implicit WTP thresholds in Sweden. The results were to a large extent driven by the relative efficacy in preventing severe exacerbations.

The results when comparing tiotropium to usual care are in line with those found in Hettle et al. [28], from which some of the data used in this study has been obtained. Hettle et al. estimated a cost per QALY gained of around £16,000 (SEK 170,000) in a UK setting in 2012. Considering the different cost structures and patient populations in UK and Sweden, the number is not too far astray from the cost per QALY gained of SEK 224,850 found in this study. From a broader perspective, the results found in this study are in line with overall results found in other cost-effectiveness studies of bronchodilators. Mauskopf et al. [38], a systematic review of 17 cost-effectiveness studies, found that tiotropium monotherapy was either cost-saving or highly cost-effective compared to other non-LAMA bronchodilator monotherapies. Similarly, Van Mölken et al. [22], a systematic review of 40 cost-effectiveness studies, concluded that all studies assessing tiotropium treatment, reported beneficial health effects of tiotropium versus placebo, ipratropium or salmeterol, sometimes even at a reduction in total COPD-related healthcare costs. In light of these findings and the findings of this study this suggests that tiotropium is highly cost-effective compared to usual non-LAMA care also in Sweden.

When comparing tiotropium monotherapy to other LAMA monotherapies, e.g. glycopyrronium, there are few published cost-effectiveness studies at this point to compare results to. Efficacy estimates from GLOW1-2 and SPARK studies suggest that tiotropium and glycopyrronium are equivalent in terms of overall lung function (FEV_1_). Thus, cost-effectiveness is likely to be highly dependent on the ability to prevent exacerbations, particularly severe ones that lead to costly hospitalisations. Using the results from the SPARK study which was favourably designed to detect exacerbations, this study has shown that, given the best available evidence on exacerbation prevention to date (SPARK), the balance would likely sway in favour of tiotropium being a more cost-effective alternative to glycopyrronium.

Extensive sensitivity analyses were conducted to assess the impact of key parameters on the main results. The relative efficacy of the interventions, particularly in preventing severe exacerbations, played an important role in explaining cost-effectiveness. In this regard, it is important to remember the basic design and purpose of the UPLIFT and SPARK trials and how these factors potentially affected the results. The purpose of the UPLIFT trial was to assess the effect of the interventions on overall lung function; exacerbations were only a secondary objective. Additionally, the patients received usual non-LAMA care in both arms. This included LABA and/or corticosteroid therapy in a majority of the patients enrolled. The trial not being primarily designed to detect exacerbations and the use of alternative inhaled therapy, likely affected rates of exacerbations downwards. This may explain why the study found no significant difference in the rates of severe exacerbations between the two arms.

As opposed to the UPLIFT trial, the SPARK trial was designed specifically to detect moderate and severe exacerbations. High risk patients, i.e. those having had at least one moderate exacerbation in the past year, were enrolled and randomly assigned to receive either QVA149 (LAMA/LABA combination), tiotropium or glycopyrronium. Importantly, patients that were receiving LABA therapy, discontinued the therapy upon enrolling in the trial, although ICS therapy continued. Indeed, these circumstances suggest that the SPARK trial was favourably designed to detect and record exacerbations. Having said that, there were only 364 severe exacerbations reported in total across all three arms in SPARK. The low number of observed severe exacerbations naturally introduces some uncertainty in the estimate of the relative risk between tiotropium and glycopyrronium and therefore also increases the uncertainty of cost-effectiveness results. It is however important to keep in mind that, as opposed to mild and moderate exacerbations, severe exacerbations are infrequent events in COPD. In addition, SPARK did not include a placebo arm, which means that all arms in the trial contained active therapy with the specific aim of lowering rates of exacerbations. These circumstances suggest that even if a trial is favourably designed to detect severe exacerbations, it is always going to be difficult to amass a large study population to record enough severe exacerbations. Future research should nevertheless continue to focus efforts on disentangling the relative efficacy of different types of bronchodilators in preventing these rare events, as they are important factors explaining morbidity, mortality and cost-effectiveness in COPD.

Finally, a potential drawback with this study, like any other utilising trial data, is that the patient population might be somewhat different from the actual population of COPD patients (GOLD II–IV) in Sweden. Measures, such as incorporating baseline mortality rates for Sweden, were taken to limit this potential bias.

## Conclusion

Using data from UPLIFT and SPARK studies, and explicitly modelling the effects of exacerbations, the cost-effectiveness of tiotropium when added to usual (non-LAMA) care was assessed relative to usual (non-LAMA) care alone. A second comparison assessed the cost-effectiveness of tiotropium relative to glycopyrronium (another LAMA). Tiotropium was deemed to be highly cost-effective relative to both comparators at current willingness-to-pay thresholds in Sweden. The findings were mainly driven by tiotropium being a relatively efficacious intervention in preventing exacerbations, particularly severe ones that lead to hospitalisations.

## Abbreviations

COPD: chronic obstructive pulmonary disease
Ex: non-severe exacerbation (mild or moderate)
FASS: database with information on approved drugs in Sweden
FEV_1_: forced expiratory volume in 1 second
FVC: forced vital capacity
GOLD II: GOLD classification indicating moderate COPD
GOLD III: GOLD classification indicating severe COPD
GOLD IV: GOLD classification indicating very severe COPD
GLY: glycopyrronium
GOLD: global initiative for chronic obstructive lung disease
ICER: incremental cost-effectiveness ratio
ICS: inhaled corticosteroid therapy
LABA: long-acting β_2_-agonists
LAMA: long-acting anticholinergic
PSA: probabilistic sensitivity analysis
QALY: quality adjusted life year
QoL: quality of life
QVA149: glycopyrronium and indacaterol combination
RR: relative risk
SEK: Swedish krona
SGRQ: St. George’s respiratory questionnaire
SevEx: severe exacerbation (requiring hospitalisation)
TIO: tiotropium
TLV: Dental and pharmaceutical benefits agency
UC: usual (non-LAMA) care
WTP: willingness-to-pay
